# A multi-scale pooling convolutional neural network for accurate steel surface defects classification

**DOI:** 10.3389/fnbot.2023.1096083

**Published:** 2023-02-14

**Authors:** Guizhong Fu, Zengguang Zhang, Wenwu Le, Jinbin Li, Qixin Zhu, Fuzhou Niu, Hao Chen, Fangyuan Sun, Yehu Shen

**Affiliations:** ^1^School of Mechanical Engineering, Suzhou University of Science and Technology, Suzhou, China; ^2^College of Mechanical and Electrical Engineering, Shihezi University, Shihezi, China

**Keywords:** convolutional neural network, multi-scale, defect classification, class activation map, feature visualization

## Abstract

Surface defect detection is an important technique to realize product quality inspection. In this study, we develop an innovative multi-scale pooling convolutional neural network to accomplish high-accuracy steel surface defect classification. The model was built based on SqueezeNet, and experiments were carried out on the NEU noise-free and noisy testing set. Class activation map visualization proves that the multi-scale pooling model can accurately capture the defect location at multiple scales, and the defect feature information at different scales can complement and reinforce each other to obtain more robust results. Through T-SNE visualization analysis, it is found that the classification results of this model have large inter-class distance and small intra-class distance, indicating that this model has high reliability and strong generalization ability. In addition, the model is small in size (3MB) and runs at up to 130FPS on an NVIDIA 1080Ti GPU, making it suitable for applications with high real-time requirements.

## 1. Introduction

Surface defect detection is one of the most important processes that affects the quality of the products (Ravikumar et al., 2011). Some surface defects will not only affect the appearance of the product surface but also endanger the user's property and life safety of users. In the beginning, surface defect detection is realized by manual inspection, hindering the improvement of productivity. It is vital to develop competent defect detection systems to replace manual work and satisfy the growing demands for automated inspection in the manufacturing sector (Song and Yan, 2013; Neogi et al., 2014).

With the development of machine vision technology, defect detection task has attracted extensive attention from researchers in the industry. A typical visual inspection system includes hardware and defects identification algorithms. These algorithms use different kinds of approaches to implement defect detection, template-based (Song and Yan, 2013), morphological filter (Mak et al., 2009), Fourier transforms (Zorić et al., 2022), Gabor filters (Bissi et al., 2013), wavelet (Li and Tsai, 2012; Li et al., 2015), Markov random field (Dogandžić et al., 2005), sparse dictionary reconstruction (Kang and Zhang, 2020), decision tree (Aghdam et al., 2012), random forest (Zhang et al., 2019), and support vector machines (Chu et al., 2017). These methods achieve the representation of defect features through manually designed feature extractors, which are highly subjective, and their defect recognition performance is affected by the designer. The detection performance will be somewhat compromised as the defect's morphology changes and the generalization ability of these detection approaches are limited.

In recent years, the theory of artificial neural networks and graphic processing unit (GPU) has been developed rapidly. Convolutional neural network (CNN) brings a new solution to vision-based tasks such as object classification and detection. LeNet is proposed by LeCun et al. (1998), it is the first CNN that can be applied to handwriting character recognition on letters. AlexNet is the next influential CNN model, it is the first CNN deployed on GPU, which can greatly improve the speed of training and testing, and provides a research basis for the subsequent extensive application of the CNN model (Krizhevsky et al., 2012). The next representative model is VGG (Simonyan and Zisserman, 2015), it incorporates 3 × 3 kernel to reduce parameters, and it is deeper and better than the previous model. The VGG16 model achieves 92.7% top-five test accuracy in ImageNet. He Kaiming et al. proposed ResNet (He et al., 2016), a very deep neural network with hundreds of layers, and skip connections are used to jump over some layers to enhance gradient backpropagation and restrain gradient vanish. Afore-mentioned models tend to use more stacked layers to obtain higher classification accuracy, which leads to an increasing number of network parameters, reducing the computation efficiency. To solve this problem, some researchers have proposed a CNN model with fewer parameters and high accuracy. Iandola et al. proposed SqueezeNet (Iandola et al., 2016), which incorporates 1 × 1 and 3 × 3 kernels to build the model, it is about 1/50 parameters of AlexNet. Howard et al. proposed a lightweight deep neural network-MobileNet (Howard et al., 2017) which could be applied to mobile and embedded vision applications.

With the emergence of constantly updated image classification models, CNN has been applied to various fields, such as medical image processing (Wang et al., 2018, 2020). Surface defect classification of industrial products is also an important application of CNN. Khumaidi et al. proposed a CNN model to obtain welding defect classification (Khumaidi et al., 2017). Li et al. proposed an end-to-end surface defects recognition system that incorporates a defect saliency map and convolutional neural network (Li et al., 2016). Fu et al. proposed a deep-learning-based model, which emphasizes the training of low-level features and incorporates multiple receptive fields (Fu et al., 2019). Ren et al. presented a CNN model to perform surface defects inspection task, and feature transferring from pre-trained models is used in the model (Ren et al., 2018). At present, in most of the research articles on defect detection, the feature distribution of the testing set and training set is relatively consistent, which cannot effectively test the generalization ability of the model. In the actual model deployment process, there are differences between the collected images and testing set, and the generalization ability of the model should also be considered an important aspect of model performance evaluation. In addition, the contents of current defect detection research papers mainly focus on the accuracy and performance comparison, and there are few defects features and rules studied by researchers, which are insufficient to establish clear corresponding relationship between the internal features of neural networks and defect detection tasks.

To settle the two problems, we propose a lightweight CNN model to achieve precise and efficient steel surface defect classification. Our CNN model is constructed on the SqueezeNet pre-trained model, an innovative multi-scale pooling (MSP) module which is proposed to learn semantic features at different scales. These features pooled on the three dimensions have been jointly considered to predict an optimal classification result. To explore the hyperparameter characteristics and their distribution rules obtained through training the model, the class activation map is used to visualize the activated features. Furthermore, to analyze the distribution information of the high-dimensional features in the CNN model, T-distribution and stochastic neighbor embedding (T-SNE) are used to reduce the data dimension to obtain a more comprehensible class-specific feature distribution rule.

Our proposed model runs about 130 FPS on a single NVIDIA 1080Ti GPU (12G memory), which can meet the needs of manufacturing enterprises for defect identification efficiency. Overall, the main contributions of this study are summarized as follows:

- We propose a lightweight CNN model, which is constructed based on SqueezeNet. An architecture that integrates class-specific defect cues after pooling rich features at multiple scales is proposed.
- The proposed model is initialized using the SqueezeNet pre-trained model, it is then fine-tuned by transferring learning across the NEU dataset. The model is tested on the noise dataset to confirm the generalization ability of the proposed model.
- The distribution characteristic of the defect at multi-scale learned by the neural network is analyzed using the class activation map, which sheds insight into the position of the defect features that are crucial for classifying the defect type. T-SNE is used to analyze the classification feature vectors in neural networks to further demonstrate the generalization ability of the proposed model.

The rest of the article is organized as follows. In Section 2, the SqueezeNet model and an optimization module are introduced. The performance and experimental comparisons of our proposed model are presented in Section 3. Finally, Section 4 concludes the article.

## 2. Proposed method

In this section, the details of pre-trained model and a multi-scale pooling module are presented. The complete structure of the proposed model is shown in **Figure 2**.

### 2.1. SqueezeNet-based defect classification

**Figure 1:**
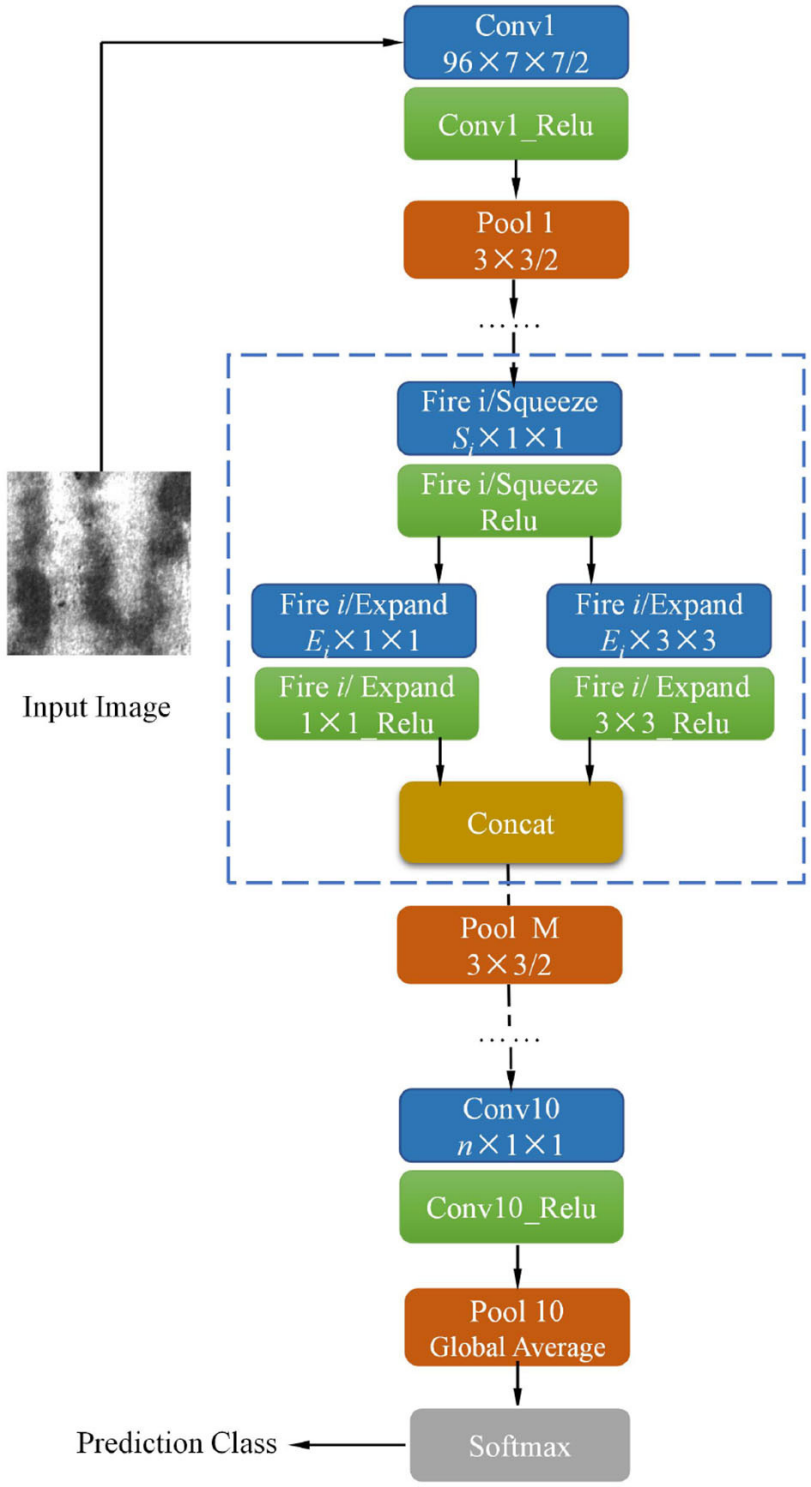
The architecture of the pre-trained SqueezeNet model (Iandola et al., 2016).

In the past decade, much of the research on convolution neural networks (CNNs) has focused on image classification task. Researchers have found that deepening the network depth can effectively improve the classification accuracy (He et al., 2016), as a result, increasing the CNN depth has become an important technique to improve performance. With the improvement of the network depth, the parameters in the network and the computational burden are also increasing. However, some industrial product inspection task requires real-time speed, so it is extremely important to develop a model that can accurately and quickly identify the image. The lightweight SqueezeNet (Iandola et al., 2016) can achieve high accuracy without loss of efficiency. The whole structure schematic diagram of the SqueezeNet model is shown in Figure 1.

In previous models, such as AlexNet (Krizhevsky et al., 2012) and VGG (Simonyan and Zisserman, 2015), the convolutional layers are constructed entirely using 3 × 3 filters. Compared to AlexNet and VGG, SqueezeNet has two advantages (1) Replace of 3 × 3 filters with 1 × 1 filters, (2) Decrease the number of input channels of 3 × 3 filters.

We plan to build our model based on the SqueezeNet pre-trained model. Pre-trained model is helpful for improving model performance in machine/computer vision-related tasks. Traditional non-deep-learning-based (machine learning-based or statistical) methods use hand-crafted features (Mak et al., 2009; Bissi et al., 2013; Song and Yan, 2013; Zorić et al., 2022). Researchers can observe the dominant feature regularity in a small amount of data to design feature extractors. However, for deep-learning-based models, the model learns the characteristic distribution of the features from a large amount of data. In general, the higher the complexity of the model, the more data are required. Inadequate data may lead to problems such as overfitting and weak generalization ability (Belkin et al., 2019; Xu et al., 2020). In the industrial defect inspection task, it is difficult to obtain a large number of images for there are limited specimens. However, transfer learning provides a feasible scheme to alleviate this problem, where a model is trained on ImageNet at first and then fine-tuned on the target dataset. The effectiveness of transfer learning methods based on pre-trained models has been demonstrated on a large number of machine/computer vision-related tasks (Lu et al., 2020; Bouaafia et al., 2021). The surface defect inspection task is an important application of machine/computer vision in the industrial field, so it is reasonable and effective to apply the pre-trained model to our task. In addition, the pre-trained models like VGG, ResNet, and SqueezeNet are publicly available (Simonyan and Zisserman, 2015; He et al., 2016). There is no need for the researcher to train the model on ImageNet again. Therefore, we build the proposed convolutional neural network based on the SqueezeNet pre-training model.

There are two individual convolutional layers ( Conv1 and Conv10), three pooling layers, and nine fire modules. Each fire module is comprised of a squeeze layer (namely Fire *i*/ Squeeze) and two expand layers (namely Fire *i*/ Expand 1 × 1 and Fire *i*/ Expand 3 × 3), among which *i* is the sequence number of fire module. The detailed configurations of nine fire modules and pooling layers are presented in Table 1.

**Table 1:** Detailed parameter configurations of layers in the SqueezeNet model.

**Layer name**	***i***	***S*_*i*_**	***E*_*i*_**
Fire 2	*i* = 2	16	64
Fire 3	*i* = 3	16	64
Fire 4	*i* = 4	32	128
Pool 4	–	–	–
Fire 5	*i* = 5	32	128
Fire 6	*i* = 6	48	192
Fire 7	*i* = 7	48	192
Fire 8	*i* = 8	64	256
Pool 8	–	–	–
Fire 9	*i* = 9	64	256

The stride of pooling layers Pool 1, Pool 4, and Pool 8 is set to 2. It can be observed that the third pooling layer, Pool 8, appeared in the deep location of SqueezeNet, ensuring the feature maps (Fire 5 ~ Fire 8) have high resolution. In each fire module, the channel number of the squeeze layer is *S*_*i*_, expand layer *E*_*i*_, *S*_*i*_ is set to a quarter of *E*_*i*_ in all fire modules to reduce the number of input channels to 3 × 3 expand layer. The pre-trained model is trained in ImageNet; *n* is the channel number of “Conv 10” as shown in Figure 1, which is set to 1,000 originally. To apply SqueezeNet to our task, *n* of “Conv 10” is modified to 6 because there are six types of defects in the NEU datasets (Song and Yan, 2013). A global average pooling (GAP) layer “Pool 10” is then connected to Conv10. GAP computes the average value of the input feature map. GAP could partially retain the spatial structure information of the input feature. Furthermore, compared to the traditional fully connected layer, the GAP does not generate additional parameters thus reducing the computational burden. The final layer is the Softmax loss layer, which is used to compute the cross-entropy between network output and label. Specifically, the loss function is defined as


(1) $$\begin{array}{c} Loss=-\overset{n}{\underset{k=i}{\sum }}{\hat{t}}_{i}\log f\left(z_{i}\right), \end{array}$$

where *n* = 6, ${\hat{t}}_{i}=1$ when the label of input image is *i*, otherwise ${\hat{t}}_{i}=0$. *f*(*z*_*i*_) is the confidence score, which is calculate by softmax, defined as


(2) $$\begin{array}{c} f\left(z_{i}\right)=\frac{e^{z_{i}}}{\overset{n}{\underset{j=1}{\sum }}e^{z_{j}}}, \end{array}$$

where *z*_*i*_ is the number *i* output value of “Pool 10.”

### 2.2. Model Optimization

**Figure 2:**
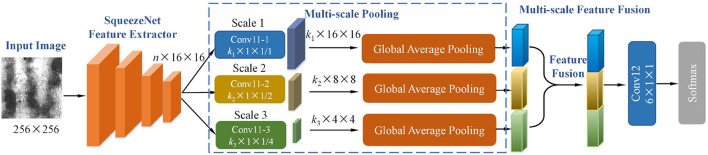
The architecture of the proposed multi-scale pooling convolutional neural network.

Based on the constructed Squeezenet model, this study proposes a multi-scale pooling (MSP) and multi-scale feature fusion structure to improve the accuracy of defect classification. The whole architecture of the proposed model is shown in Figure 2. All the activation functions used in our model are Rectified Linear Unit (Relu). All the activation functions are omitted for brevity, as shown in Figure 2.

The layers from Conv1 to fire 9 in the SqueezeNet model are used as a feature extractor to extract defect features of an input image. The input image size is 256 × 256. After feature extraction based on SqueezeNet, the output feature dimension is *n*×16 × 16, where *n* is as defined earlier. In the multi-scale pooling module, three different convolutional layers are connected to the feature extractor separately. The detailed parameters of three convolutional layers: (1) Conv11-1 uses *k*_1_×1 × 1 filter, stride = 1; (2) Conv11-2 uses *k*_2_×1 × 1 filter, stride = 2; (3) Conv11-3 uses *k*_3_×1 × 1 filter, stride = 4. After convoluted by Conv11, the dimension of output feature maps at three scales are *k*_1_×16 × 16, *k*_2_×8 × 8, and *k*_3_×4 × 4. We set *k*_1_ = 6, *k*_2_ = 6, and *k*_3_ = 6 for six classes of defect, which is helpful to promote the convolutional layer *Conv*11 to learn better class-specific features. Considering there are different types of defects, the defects differ in pattern size and morphology. The proposed multi-scale module in our model could effectively capture semantic defect features at different scales. The detailed visual comparison of the defect recognition effect of the model at different scales will be shown in Section 3.5. The afore-mentioned process is described as


(3) $$\begin{array}{c} h_{j}^{m_{i}\left(j\right)}\left(x\right)=f\left(w_{j}^{m_{i}\left(j\right)}x+b_{j}^{m_{i}\left(j\right)}\right) \end{array}$$

where *x* is the output of SqueezeNet feature extractor, $w_{j}^{m_{i}\left(j\right)}$ and $b_{j}^{m_{i}\left(j\right)}$ the weight and bias of the *m*_*i*_(*j*)*th* channel filter in Conv11−*j* layer (*j* is the scale number, and *j* = 1, 2, 3), $h_{j}^{m_{i}\left(j\right)}$ is the *m*(*j*)*th* channel filter output after processed by Conv11−*i*, *m*_*i*_(*j*) = 1~*k*_*j*_.

Global average pooling layer calculate the average value of input tensors ($h^{m_{i}\left(j\right)}$) across *k*_*j*_ channels, each channel generates a class-related value (Lin et al., 2013). The operation is described as


(4) $$\begin{array}{c} y_{j}^{m_{i}\left(j\right)}=\frac{1}{R}\underset{\left(p,q\right)\in R}{\sum }h_{jpq}^{m_{i}\left(j\right)} \end{array}$$

where $y_{j}^{m_{i}\left(j\right)}$ is the *m*_*i*_(*j*)*th* feature map output value in scale *j*, *R* is the element total number of *m*_*i*_(*j*)*th* feature map, and $h_{jpq}^{m_{i}\left(j\right)}$ is the element value at (*p, q*) in region *R*.

In the multi-scale feature fusion stage, the output of three global average pooling layers are stacked together by a concat layer, which defined as


(5) $$\begin{array}{c} F_{y}=f^{cat}\left(Y_{1},Y_{2},Y_{3}\right) \end{array}$$

where *Y*_*j*_ is the full set of $y_{j}^{m_{i}\left(j\right)}$, defined as $Y_{j}=\left\{y_{j}^{m_{i}\left(j\right)}\right\}$, *f*^*cat*^ represents the concatenation operation stacking pooled values *Y*_1_, *Y*_2_, and *Y*_3_ together. *F*_*y*_ is the fused tensor, due to *m*_*i*_(*j*) = 1~*k*_*j*_, total length of *F*_*y*_ is *k*_1_+*k*_2_+*k*_3_. *F*_*y*_ is finally convoluted by a convolution layer, using six channel number and 1 × 1 kernel size. The parameters in feature extractor and new-added layer are updated by cross-entropy loss, which is already introduced in Section 2.1.

Liu et al. proposed a lightweight model with multi-scale features for steel surface defect classification (Liu et al., 2020). Their model is named ConCNN, which is a concurrent CNN including input of two different image scales. Specifically, the 200 × 200 and 400 × 400 pixel images are input into two independent sub-networks with the same structure, and the output feature vectors of both sub-networks are fused to obtain the final classification output. Our proposed model is quite different from the ConCNN, and the difference includes two significant aspects. First, the multi-scale features in our model are achieved by constructing different pooling layers at the high level of the network; ConCNN inputs two scales of images so that two individual sub-networks learn features at two scales. Second, our model uses a pre-trained model to initialize the parameters in the network, while the parameters of ConCNN are randomly initialized. Yu et al. (2021) applied the high-order pooling for Alzheimer's disease assessment. The high-order pooling module is incorporated into the classifier to make full use of the correlation within feature maps along the channel axis to capture more discriminative CNN features. Different from high-order pooling, the proposed multi-scale pooling is constructed using first-order pooling at different scales. The focus of the two types of pooling is different.

In our proposed model, the output of the SqueezeNet feature extractor passes through the convolution layer with large step size; the most significant defect features will be retained, while small-size defect features will be suppressed in this process. More detailed features will be preserved when processed by the convolution layer with a small step size. The suggested model has a good detection performance because it can collect defect characteristics of both large and small sizes at a high level by combining the defect feature information from both sources. The effectiveness of the proposed multi-scale pooling structure is systematically evaluated in Section 3.3.

## 3. Experiments

### 3.1. Training and testing database

**Figure 3:**
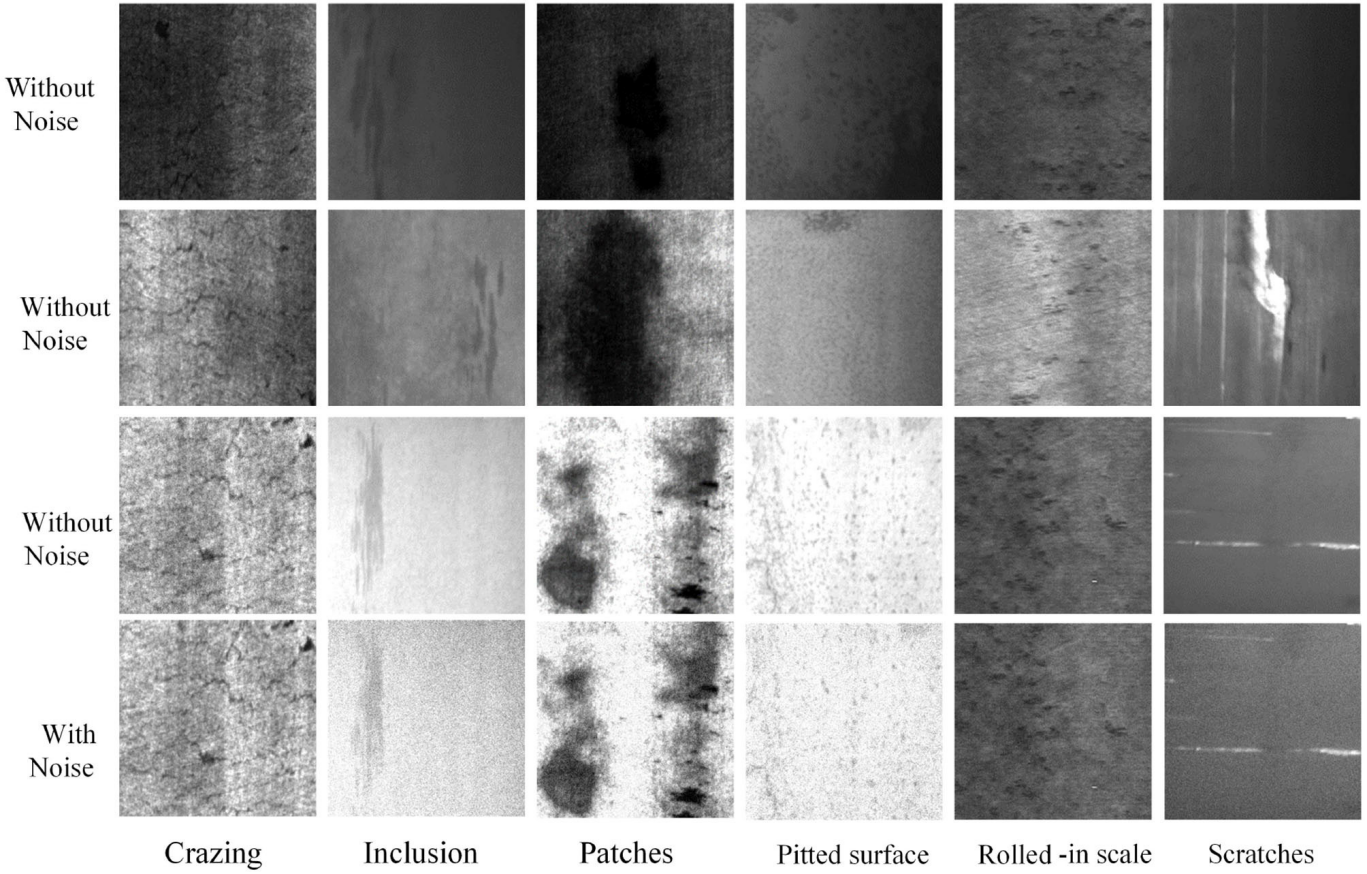
Samples images of six classes of surface defects in the NEU surface defect dataset and a corresponding noise testing dataset.

The NEU steel surface defect dataset (Song and Yan, 2013), which is freely accessible online, is the basis for our experiment. There are six classes of defects in the dataset, Crazing (Cr), Inclusion (In), Patches (Pa), Pitted surface (Ps), Rolled-in scale (Rs), and Scratches (Sc). Some sample images of different defect classes are shown in the top three rows of Figure 3. It can be seen that the morphology of different defect types can show great differences, including color, texture features, and defect size, bringing challenges for defect classification. For each defect class, there are 300 images. The dataset is divided into the training set and the testing set. A total of 80% of samples is selected as the training set and 20% as the testing set. We do not make use of any data augmentation techniques in the training and testing sets.

Image noise, which is typically brought on by electronic noise or environmental variables, is a random variation of color values in acquired photographs. A typical type of picture noise, Gaussian noise, is applied to the testing set to examine the effectiveness and generalization ability of the suggested strategy in the case of potential noise. Comparative samples of NEU testing set and corresponding Gaussian noise images are shown in the bottom two rows of Figure 3.

Gaussian noise is statistical noise in which probability density function is Gaussian distribution. The Gaussian distribution is defined as


(6) $$\begin{array}{c} p\left(t\right)=\frac{1}{\sqrt{2\pi }\sigma }e^{-\frac{{\left(t-\mu \right)}^{2}}{2\sigma^{2}}} \end{array}$$

where μ is average value and σ is standard deviation. To control the intensity of noise, μ and σ are determined by a signal-to-noise ratio (SNR) value, which is defined as


(7) $$SNR=10\log_{10}\left[\frac{\sum_{i=1}^{H}\sum_{j=1}^{W}s{\left(i,j\right)}^{2}}{\sum_{i=1}^{H}\sum_{j=1}^{W}n{\left(i,j\right)}^{2})}\right],$$

where *H* and *W* indicate the height and width of the input image; *s*(*i, j*) and *n*(*i, j*) are the pixel values of signal and noise at pixel location (*i, j*). The SNR value is set to 20 dB. Each noise image are generated five times, and add up to 1,800 images (60 × 6 × 5). With the addition of noise, defect morphologies of six types have changed, which brings difficulties to the defect identification task.

### 3.2. Implementation Details

Caffe is one of the widely used deep-learning frameworks that are publicly available (Jia et al., 2014). All the experiments are implemented in Caffe. Stochastic gradient descent policy is used to train the CNN models with a weight decay of 10^−4^ and a momentum of 0.9. The batch size is set to 32, which means 32 sample images are computed per iteration. The basic learning rate is 0.01 and after every 800 iterations, the learning rate becomes one-tenth of the original. NVIDIA 1080Ti GPU(12GB) is used in experiments to realize parallel computing and achieve good performance. We use Xavier's initialization (Glorot and Bengio, 2010) for convolutional layers in our proposed model.

### 3.3. Comparisons with other models

In order to verify the effectiveness of the proposed MSP module and model, we compare our model with other defect classification models, including machine learning (ML)-based and CNN-based approaches. Among these approaches, the ML-based classifiers include support vector machine (SVM), nearest neighbor clustering (NNC), and multiple linear regression (MLR). Three feature extractors–Gray level co-occurrence matrix (GLCM) (Haralick et al., 1973), adaptive extended local ternary pattern (AELTP) (Mohamed and Yampolskiy, 2013), and adjacent evaluation completed local binary patterns (AECLBP) (Song and Yan, 2013)—are used. Nine classification methods can be obtained by combining different feature extractors and classifiers. Moreover, several CNN-based approaches are also compared, including ETE (Li et al., 2016), DECAF+MLR (Ren et al., 2018), AlexNet (Krizhevsky et al., 2012), ConCNN (Liu et al., 2020), and SqueezeNet (Iandola et al., 2016; Fu et al., 2019). For a fair comparison, all the approaches are trained and tested on the same training and testing set, respectively. The training set includes 1,440 sample images (240 × 6), and the testing set includes 360 sample images without noise (60 × 6) and 1,800 with noise (60 × 6 × 5).

The comparative results are shown in Table 2.

**Table 2:** The classification accuracy (%) of various steel surface defect classification approaches in both the NEU datasets without/with noise.

**Method**	**NEU**	**NEU with noise**
GLCM+SVM (Haralick et al., 1973)	88.1	67.8
GLCM+NNC (Haralick et al., 1973)	89.7	71.0
GLCM+MLR (Haralick et al., 1973)	94.7	52.3
AELTP+SVM (Mohamed and Yampolskiy, 2013)	76.1	44.6
AELTP+NNC (Mohamed and Yampolskiy, 2013)	96.4	64.4
AELTP+MLR (Mohamed and Yampolskiy, 2013)	98.6	48.8
AECLBP+SVM (Song and Yan, 2013)	98.9	39.9
AECLBP+NNC (Song and Yan, 2013)	98.3	42.7
AECLBP+MLR (Song and Yan, 2013)	98.3	43.7
ETE (Li et al., 2016)	95.8	47.6
DECAF+MLR (Ren et al., 2018)	99.7	89.7
AlexNet (Krizhevsky et al., 2012)	91.4	83.4
ConCNN (Liu et al., 2020)	99.6	84.5
SqueezeNet (Iandola et al., 2016; Fu et al., 2019)	99.7	82.9
SqueezeNet+MSP(Proposed)	100	94.6

It is noted that the classification accuracy of most machine-learning approaches is higher than 85% on the NEU dataset without noise. The defect feature characteristic of the testing set and the training set is relatively consistent. After the feature extractor obtains the defect features on the training set, the model trained by the machine learning classifier is also applicable to the testing set. The proposed model achieves 100% accuracy, better than the rest of CNN approaches (100% vs. 95.8%, 99.7%, 91.4%, 99.6% and 99.7%). For the testing set NEU with noise, the accuracy of most approaches has dropped dramatically, especially ML-based approaches—GLCM/AELTP/AECLBP+SVM/NNC/MLR. With the addition of noise, defect feature characteristics of the sample images change accordingly. As a result, those models with poor generalization ability could not identify the defect class accurately. Among the CNN-based models, our proposed model still achieved the highest accuracy (94.6%), which is much higher than DECAF+MLR (Ren et al., 2018) (89.7%), AlexNet (Krizhevsky et al., 2012) (83.4%), and ConCNN (Liu et al., 2020) (84.5%). Among these methods, ConCNN (Liu et al., 2020) could achieve a close performance to ours in the NEU dataset, but the accuracy drops to 84.5% in the noisy dataset. The reason behind this is that ConCNN constructed by inputting two scale images encountered difficulties in recognizing defect features with noise. According to the previous discussions, it can be concluded that by adding the MSP module to SqueezeNet, the accuracy improves from 82.9% to 94.6%, which proves that our proposed MSP is effective and robust.

In order to obtain more detailed defect classification information, the confusion matrixes of SqueezeNet and the proposed model on two testing sets are shown in Table 3. Due to six classes of defect type, each confusion matrix has six columns and six rows. Each column of the confusion matrix represents the prediction class, and the total number of each column represents the number of data predicted for this class. Each row represents the real class of defect image and the total number of data in each row represents the number of image samples of that class. For example, the number in the third row (Pa) and the second column (In) represents the total number of samples whose real class is Pa, which is predicted to be In. Table 3A shows the classification results of SqueezeNet in the NEU testing set, it can be seen that the only one In defect sample is wrongly identified as PS. Table 3B shows the results in the noisy set; it can be seen that the accuracy of identifying In and PS is low, indicating that they are easy to be confused with other defect types. From Table 3C, it can be seen from the experimental results that all defect classes are correctly identified on the NEU testing set without noise. Table 3D shows the results of the NEU testing set with noise, the defect samples Cr, Pa, and RS are 100% accurately identified, In is easily confused with PS and Sc and Ps is easily confused with Cr. The experimental results prove that the proposed MSP module is helpful to improve the accuracy of defect classification.

**Table 3:** The confusion matrix of SqueezeNet (Iandola et al., 2016; Fu et al., 2019) and the proposed model on NEU steel surface defect dataset without/with noise.

**(A)**							**(B)**						
	**Cr**	**In**	**Pa**	**PS**	**RS**	**Sc**		**Cr**	**In**	**Pa**	**PS**	**RS**	**Sc**
Cr	60	0	0	0	0	0	Cr	300	0	0	0	0	0
In	0	59	0	1	0	0	In	2	133	0	41	72	52
Pa	0	0	60	0	0	0	Pa	1	0	299	0	0	0
PS	0	0	0	60	0	0	PS	106	5	14	169	5	0
RS	0	0	0	0	60	0	RS	4	0	0	0	296	0
Sc	0	0	0	0	0	60	Sc	0	0	0	0	4	296
**(C)**							**(D)**						
	**Cr**	**In**	**Pa**	**PS**	**RS**	**Sc**		**Cr**	**In**	**Pa**	**PS**	**RS**	**Sc**
Cr	60	0	0	0	0	0	Cr	300	0	0	0	0	0
In	0	60	0	0	0	0	In	0	267	0	20	0	13
Pa	0	0	60	0	0	0	Pa	0	0	300	0	0	0
PS	0	0	0	60	0	0	PS	46	3	4	239	8	0
RS	0	0	0	0	60	0	RS	0	0	0	0	300	0
Sc	0	0	0	0	0	60	Sc	0	0	2	0	0	298

**(A)** SqueezeNet in NEU testing set; **(B)** SqueezeNet in NEU testing set with noise; **(C)** proposed model in NEU testing set; and **(D)** proposed model in NEU testing set with noise.

In order to comprehensively analyze and propose the model, the running speed and model size of different CNN-based defect classification methods are also compared. The comparison results are shown in Table 4.

**Table 4:** The running time and model size of several CNN-based methods.

**Method**	**Running time(s)**	**Model size (MB)**	**Accuracy in noisy testing set (%)**
ETE (Li et al., 2016)	0.005	1.9	47.6
DECAF+MLR (Ren et al., 2018)	0.015	244	89.7
AlexNet (Krizhevsky et al., 2012)	0.085	15	83.4
SqueezeNet (Iandola et al., 2016)	0.007	3.0	82.9
Proposed	0.007	3.0	94.6

The running speed is the time for the model to process an image sample, and three repeated experiments are used to calculate the average speed. It is observed that the file size of the proposed model is 3 MB, which is easy to deploy on mobile devices. Although ETE's model runs faster, it is less accurate than the proposed model. The running speed of the proposed model reaches 130FPS on 1080TI GPU, which is able to fully satisfy the demand for fast detection in industrial scenarios.

### 3.4. Ablation study

In the proposed model, the scale parameter settings of MSP will impact the classification performance. To determine the optimal scale parameters, the following comparison experiments are conducted. In a pooling layer, the value of the stride is usually set to 2^*n*^, that is, 1, 2, 4, 8, etc. Considering the output feature width of the SqueezeNet feature extractor is 16. As a result, all the optional strides are 1, 2, 4, 8, and 16. The MSP module contains five different implementation ways, as shown in Table 5.

**Table 5:** The classification accuracy (%) of MSP using different scales in the NEU dataset without/with noise.

**The strides in MSP**	**NEU**	**NEU with noise**
1	1	0.871
1, 2	1	0.898
1, 2, 4	1	0.946
1, 2, 4, 8	1	0.932
1, 2, 4, 8, 16	1	0.930

The experimental results show that the combination of the three scales achieves the best classification accuracy. When the scale increases from one to three, the accuracy increases, and the classification accuracy declines when the number of scales increases further. This phenomenon of decreased accuracy is due to the fact that the pooling layer with large strides will lose too much location information, which is detrimental to defect feature recognition. Therefore, the proposed MSP in our study is composed of three pooling layers using 1, 2, and 4 strides, respectively.

### 3.5. Class activation map analysis

**Figure 4:**
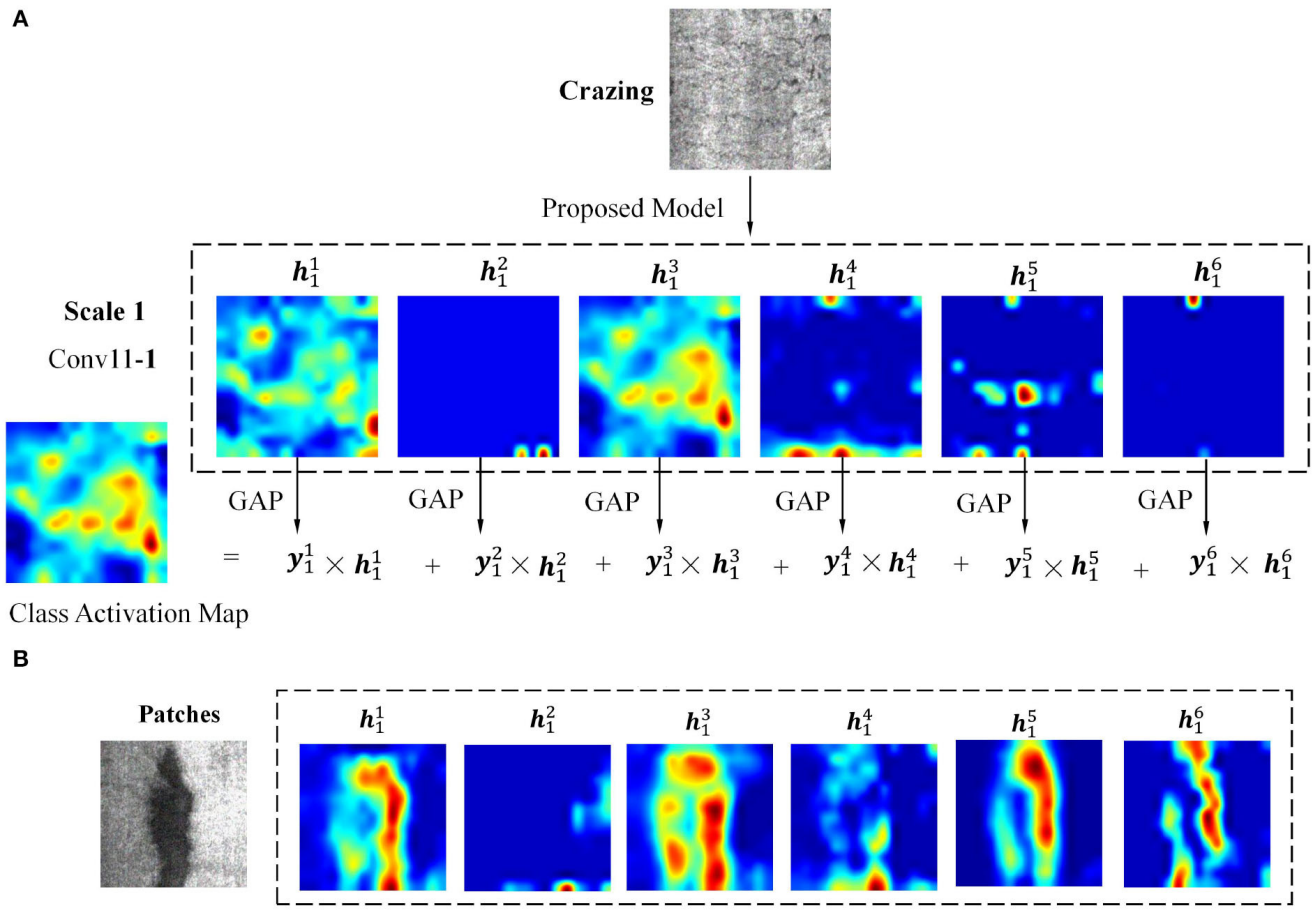
The detailed calculation process of class activation map at scale 1. **(A)** Test sample Crazing; **(B)** Test sample Patches.

In order to analyze the defect features learned in neural networks and which defect features are the key to judge the defect type, the class activation map (CAM) is used for feature analysis in neural networks (Zhou et al., 2016). In the proposed multi-scale pooling module, conv11−*i* are the last convolutional layers, and defect features are learned at three scales. By multiplying the feature map in conv11−*i* and the output value of the corresponding global average pooling layer, summing up all products, the CAM is obtained. The process is calculated as


(8) $$\begin{array}{c} M_{j}=\overset{k_{j}}{\underset{m_{i}\left(j\right)=1}{\sum }}h_{j}^{m_{i}\left(j\right)}\left(x\right)*y_{j}^{m_{i}\left(j\right)} \end{array}$$

where $h_{j}^{m_{i}\left(j\right)}\left(x\right)$ and $y_{j}^{m_{i}\left(j\right)}\left(x\right)$ are the feature map and corresponding activated score at *jth* scale, detailed description is given in Section 2.2. *M*_*j*_ is the class activation map at scale *j*. To explain the calculation process more intuitively, two types of defect sample images are selected as examples. The detailed calculation process of CAM at scale 1 is shown in Figure 4, where two types of defects were selected for analysis.

Figure 4A shows the calculation process of using Crazing as a testing image. In the Conv11 − 1 convolution layer, there are six feature channels, namely $h_{1}^{1}\sim h_{1}^{6}$. The six feature maps are feed into the GAP to get six activation scores, namely $y_{1}^{1}\sim y_{1}^{6}$. Specifically, the six scores are 10.588, 0.005, 49.979, 5.6444, 1.007, and 0.049, which are calculated by the intensity of pixels in the feature image. The CAM at this scale is obtained by multiplying $y_{1}^{1}h_{1}^{1},\cdots \;,y_{1}^{6}h_{1}^{6}$.

It is worth noting that some channels have richer feature composition($h_{1}^{1}$ and $h_{1}^{3}$), while others have fewer($h_{1}^{2}$ and $h_{1}^{6}$). When another defect type Pa is selected as the input image, the corresponding feature maps are shown in Figure 4B. Different from Figure 4A, $h_{1}^{1}$, $h_{1}^{3}$, $h_{1}^{5}$, and $h_{1}^{6}$ have richer feature composition, while $h_{1}^{2}$ has fewer. The explanation is that different types of defect features will be activated on different channels.

**Figure 5:**
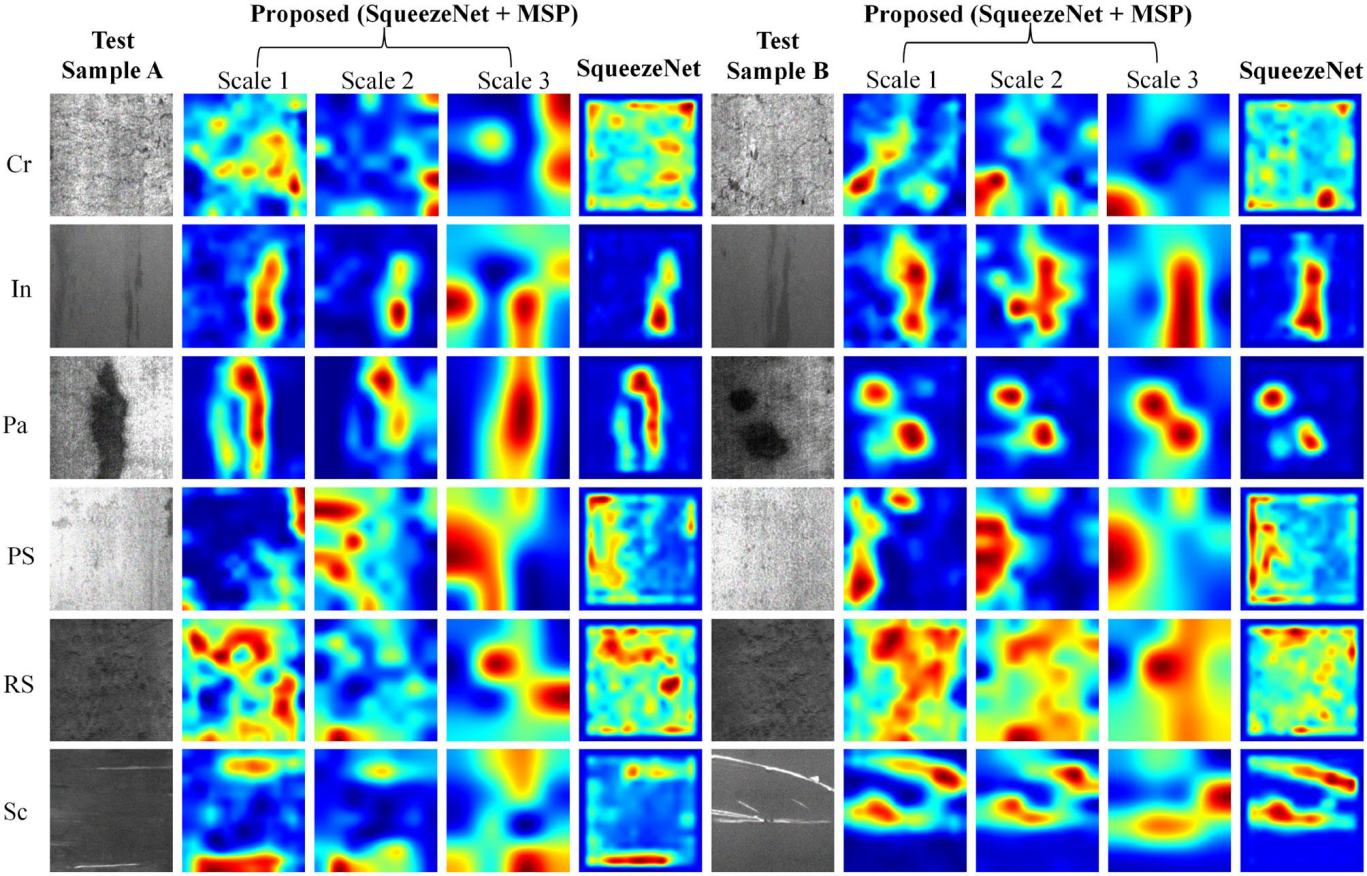
The class activation map visualization results of convolutional features in MSP module and SqueezeNet.

For a comprehensive analysis, the class activation map visualization results of six defect types at three scales in the multi-scale pooling module are shown in Figure 5. For better visual effects, the pixels of all CAM results are adjusted to 200 × 200, and the grayscale images are converted to heat map mode. Two samples of each defect type in NEU with noise testing set were selected for analysis, namely test sample A and B. To study the CAM characteristic at three different scales and their differences, the CAM of three scales in the proposed MSP module are given. To verify the effectiveness of the proposed MSP module, the MSP of SqueezeNet model is also shown for comparison.

It can be observed that the CAM at three scales can highlight the defect regions, but the focus is different. In general, the results of scale 1 have a higher resolution and can retain more detailed defect cues; scale 3 highlights the main defect regions and ignores some small-scale defect cues, while scale 2 is characterized by a synthesis of scales 1 and 3. The focused regions at three scales complement each other, such as the test sample A of Crazing, Inclusion, Patches, Pitted surface, Rolled-in scale, and Scratches. Taking sample A of Crazing as an example, it can be seen that the highlighted areas of scale 1 are relatively scattered; the highlighted areas of scale 2 are compact and concentrated in the lower right and upper right corners; highlighted areas are concentrated in the upper right corner at scale 3.

In addition to being complementary, the highlighted areas at multiple scales may show very high consistency, such as test sample B of six defect classes. Consistency of highlight areas at multiple scales could strengthen the identification of defects. Take sample B of Inclusion as an example; the CAM at all three scales focus on long-striped defect features. In addition, comparing the CAM of the proposed model with SqueezeNet, SqueezeNet does not properly focus on the defect area, such as sample A/B of Crazing, Pitted surface, and Rolled-in scale. Missing the correct defect region will result in false detection of the defect class. The afore-mentioned comparison results could confirm the validity of the newly added MSP module. In conclusion, the CAM of the proposed model could accurately locate defect locations at multiple scales, and the highlighted areas at multiple scales can complement and reinforce each other. In the subsequent feature fusion layer, the neural network can adaptively learn the relationship between features of different scales according to the characteristics of defects to obtain more reliable defect classification cues.

### 3.6. T-SNE dimension reduction visualization Analysis

To study the class-related information learned in the hidden layer of the neural network, the t-distributed stochastic neighbor embedding (T-SNE) (Van der Maaten and Hinton, 2008) dimension reduction method is used to visualize the neural network parameters. Specifically, the six numerical parameters in Conv-12 (shown in Figure 2) are used as raw data. T-SNE shows the representation of six-dimensional data in two-dimensional, intuitively, the degree of aggregation between different defect image samples. The distance of two samples in raw data (six dimensions) is calculated as


(9) $$\begin{array}{c} p_{j|i}=\frac{\text{exp}\left(-{\left||x_{i}-x_{j}|\right|}^{2}/2\sigma_{i}^{2}\right)}{\underset{k\neq i}{\sum }\text{exp}\left(-{∥x_{i}-x_{j}∥}^{2}/2\sigma_{i}^{2}\right)} \end{array}$$

where *x*_*i*_, *x*_*j*_ is the CNN features of two samples, and $\sigma_{i}^{2}$ is variance. Then, the joint distribution *P*_*ij*_ is calculated as


(10) $$\begin{array}{c} P_{ij}=\frac{P_{j|i}+P_{i|j}}{2N} \end{array}$$

where *N* is the total number of samples in testing set. The distance of projection points in two-dimension is calculated as


(11) $$\begin{array}{c} Q_{ij}=\frac{{\left(1+||y_{i}-y_{j}|{|}^{2}\right)}^{-1}}{\underset{k\neq l}{\sum }{\left(1+||y_{k}-y_{l}|{|}^{2}\right)}^{-1}} \end{array}$$

where *y*_*i*_, *y*_*j*_ is the projection points of two samples in two-dimension. Kullback–Leibler (KL) divergence is used to measure the similarity between points at high and low dimensions to ensure that points with high similarity at high dimension also have high similarity at low dimension. KL-divergence is calculated as


(12) $$\begin{array}{c} KL\left(P||Q\right)=\underset{i\neq j}{\sum }p_{ij}\log\frac{p_{ij}}{q_{ij}} \end{array}$$

**Figure 6:**
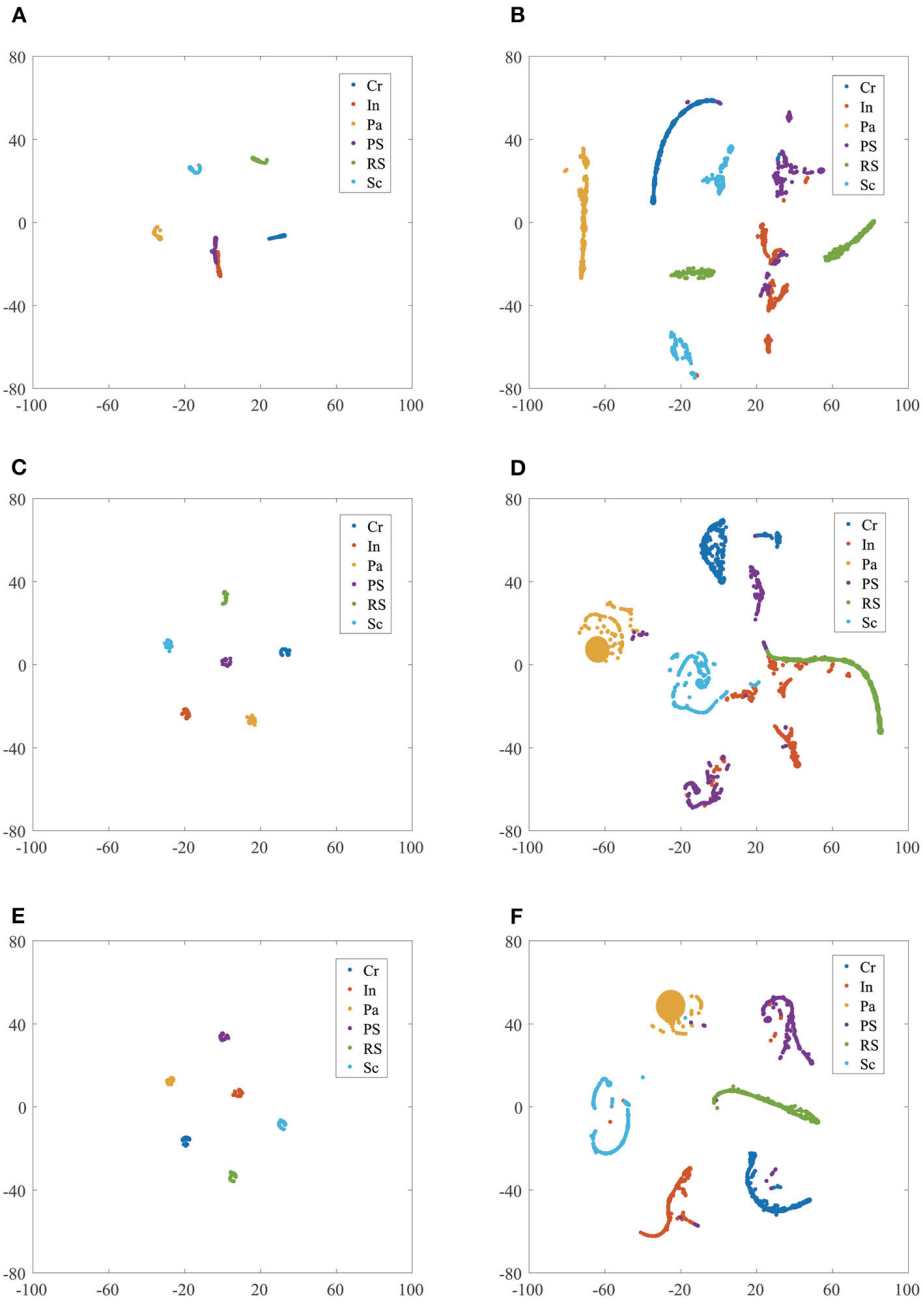
The T-SNE feature dimension reduction visualization results comparison of different CNN model classifiers in NEU dataset without/with noise. **(A)** AlexNet in NEU testing set; **(B)** AlexNet in NEU testing set with noise; **(C)** SqueezeNet in NEU testing set; **(D)** SqueezeNet in NEU testing set with noise; **(E)** proposed model in NEU testing set; **(F)** proposed model in NEU testing set with noise.

The T-SNE dimension reduction visualization results of AlexNet, SqueezeNet, and the proposed model are shown in Figure 6.

Figure 6A shows the result of AlexNet in the NEU testing set, it can be seen that the samples Cr, Pa, RS, and Sc aggregate together and have a clear separation, while In and PS mix together. Figures 6C, E shows the results of SquuezeNet and the proposed model in the NEU testing set, respectively; six classes of the defect image samples are completely separated in both. It is worth noting that the PS cluster in the proposed model is no longer in the middle of multiple classes, thus increasing the inter-class distance, which is helpful in obtaining more reliable classification results. Figures 6B, D shows the results of AlexNet and SqueezeNet in the NEU testing set with noise, respectively. It can be seen that many defect class clusters show dispersion and mix with each other, such as Pa, PS, PS, and Sc. Figure 6F shows the results of the proposed model in the NEU testing set with noise, and these clusters have a clear separation; only a few samples are mixed into other classes. To sum up, the clustering results of the proposed model have a small intra-class distance and a large inter-class distance, proving that our model has better performance and strong generalization ability.

### 3.7. Generalization ability verification

**Figure 7:**
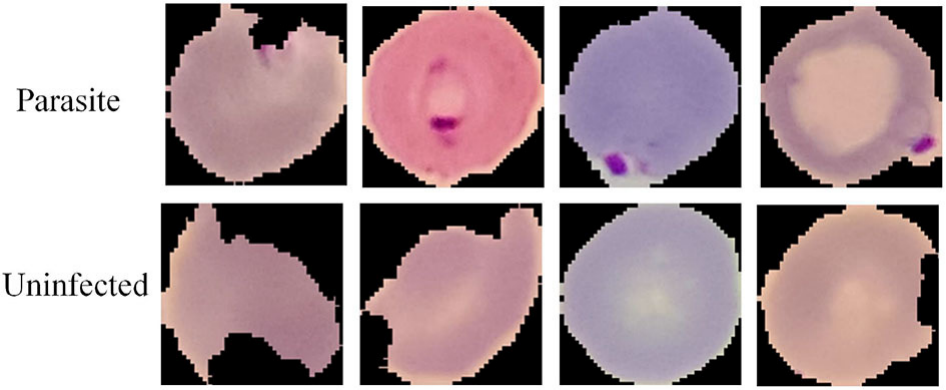
Sample images of parasitic and uninfected cell image samples in the Malaria dataset.

To verify the generalization ability of the MSP module, we conduct comparative experiments in the field of medical image processing. We chose the cell Malaria image classification task (Narayanan et al., 2019), which is provided by Kaggle. The Malaria dataset includes two classes, parasitic and uninfected, and the typical image samples are shown in Figure 7. It is observed that both cell color and shape are highly variable. The training set and the testing set have been partitioned in the original dataset and the partition of the dataset is shown in Table 6. The training set contains 220 parasitic and 196 uninfected samples, and the testing set contains 91 parasitic and 43 uninfected samples.

**Table 6:** The number of parasitic and uninfected cell image samples in the Malaria training and testing dataset (Narayanan et al., 2019).

**Malaria class**	**Parasite**	**Uninfected**
Training set	220	196
Testing set	91	43

The Malaria training set is trained on SqueezeNet and SqueezeNet+MSP model with the same training parameters, and the prediction accuracy is shown in Table 7. It can be seen that the accuracy of the SqueezeNet reaches 97.7%, and the accuracy is further improved by adding the MSP module, reaching 98.5%. This proves that the proposed MSP is effective in improving the classification accuracy of Malaria images. It can be concluded that MSP is not only applicable in the field of industrial defect detection but also in medical image processing.

**Table 7:** The classification accuracy (%) of SqueezeNet and proposed model in the Malaria dataset.

**Method**	**SqueezeNet**	**SqueezeNet+MSP (Proposed)**
Accuracy (%)	97.7	98.5

## 4. Conclusion

For the classification of steel surface defects, we propose a multi-scale pooling convolutional neural network in this research. Our model is based on SqueezeNet, and to capture defect features at various scales, we propose an innovative multi-scale pooling module. In the module, the multi-scale features are combined to produce more reliable defect cues. It is demonstrated that our model has higher accuracy by contrasting it with other defect classification models in a noisy NEU testing set. The MSP module is able to locate the defect location accurately, according to class activation map analysis, and the highlighted areas at different scales could complement and reinforce one another to produce more reliable results. According to the visualization results of T-SNE, the suggested model has a small intra-class spacing and a big inter-class spacing, which suggests a strong generalization capacity in handling noise. Furthermore, our model's 3MB size and 130 FPS performance on a single NVIDIA 1080Ti GPU, which could be applied to scenarios where device computation power is constrained and detection speed is required. In future, we intend to develop a model that can perform well on various surface defect dataset classification tasks, saving time on model fine-tuning.

## Data availability statement

Publicly available datasets were analyzed in this study. This data can be found at: http://faculty.neu.edu.cn/songkechen/zh_CN/zhym/263269/list/index.htm.

## Author contributions

All authors listed have made a substantial, direct, and intellectual contribution to the work and approved it for publication.
